# Evaluating Large Language Models for Automated Data Cleaning and Feature Engineering in Clinical Datasets

**DOI:** 10.7759/cureus.108550

**Published:** 2026-05-09

**Authors:** Shimul A Babli, Pulkit Gairola, Chukwuemeka E Ogbu, Francesco Alessi Longa, Aditya Rautaray, Opeyemi S Alamu, Syed Faheem Haider Rizvi

**Affiliations:** 1 Internal Medicine, Islami Bank Medical College and Hospital, Rajshahi, BGD; 2 Public Health, The University of Tennessee Knoxville, Knoxville, USA; 3 College of Medicine, The University of Tennessee Health Science Center, Knoxville, USA; 4 Internal Medicine, Cape Fear Valley Health, Fayetteville, USA; 5 International Law, Azteca University, Chalco, MEX; 6 Cloud Solutions Security, CVS Health, New York, USA; 7 Statistics, The Federal College of Animal Health and Production Technology, Ibadan, NGA; 8 Electronics Engineering, Sir Syed University of Engineering and Technology (SSUET), Karachi, PAK

**Keywords:** clinical data cleaning, electronic health records (ehrs), feature engineering, large language models (llms), machine learning in healthcare

## Abstract

Background: Electronic health records (EHRs) are increasingly used for clinical research and machine learning, yet they are plagued by missing values, outliers, inconsistent coding, and heterogeneous data types. Traditional rule-based cleaning pipelines demand extensive domain expertise and manual effort. Large language models (LLMs) exhibit strong code-generation and clinical reasoning abilities, but their utility for automating structured data preprocessing in clinical datasets remains underexplored.

Methods: We evaluated three LLMs, GPT-4 (OpenAI, Inc., San Francisco, United States), Claude 3.5 Sonnet (Anthropic, San Francisco, United States), and Gemini 1.5 Pro (Google DeepMind, Mountain View, United States), on five data cleaning tasks and two feature engineering tasks across three publicly available clinical datasets: Medical Information Mart for Intensive Care (MIMIC-IV) (PhysioNet), the eICU Collaborative Research Database (PhysioNet), and National Health and Nutrition Examination Survey (NHANES) 2017-2020 (CDC). LLM-generated preprocessing scripts were benchmarked against a conventional rule-based pipeline. Downstream predictive performance was assessed using XGBoost and logistic regression for in-hospital mortality prediction, evaluated by area under the receiver operating characteristic curve (AUROC) and F1 score.

Results: Claude 3.5 Sonnet achieved the highest mean data cleaning F1 score (0.90), followed by GPT-4 (0.89) and Gemini 1.5 Pro (0.85), all exceeding the rule-based baseline (0.77). For mortality prediction on MIMIC-IV, data preprocessed by Claude 3.5 Sonnet yielded the best XGBoost AUROC (0.851; 95% CI: 0.839-0.863), compared with GPT-4 (0.842), Gemini 1.5 Pro (0.829), rule-based cleaning (0.803), and no cleaning (0.761). LLM-engineered features contributed incremental AUROC gains of 0.015-0.025 over manually constructed feature sets.

Conclusions: LLMs can substantially automate clinical data cleaning and feature engineering, achieving performance comparable to or exceeding hand-crafted pipelines. However, domain expert oversight remains essential to validate clinical plausibility and prevent silent data corruption.

## Introduction

The secondary use of electronic health records (EHRs) for clinical research and predictive modeling has expanded considerably over the past decade. Large observational databases such as the Medical Information Mart for Intensive Care (MIMIC-IV) [1], the eICU Collaborative Research Database [2], and the National Health and Nutrition Examination Survey (NHANES) [3] provide unprecedented volumes of patient-level data encompassing demographics, vital signs, laboratory results, diagnoses, treatments, and clinical notes. These resources have enabled significant advances in disease risk stratification, treatment response prediction, and health system quality benchmarking, making them cornerstones of modern clinical informatics research.

Despite their scale, EHR datasets suffer from well-documented data quality challenges. Structured data quality frameworks have identified pervasive issues, including missingness rates exceeding 30% for key laboratory values, physiologically implausible outlier values, heterogeneous measurement units, inconsistent data type encoding, and duplicated records [4,5]. Conventional data cleaning approaches rely on hand-coded rules, statistical imputation libraries such as multivariate imputation by chained equations (MICE) [6] and MissForest [7], and manual expert chart review. These methods are time-intensive, institution-specific, and difficult to generalize [8]. Data preparation has been estimated to consume 60-80% of total effort in clinical machine learning projects [9], making it a significant bottleneck.

The emergence of large language models (LLMs) with strong code-generation and reasoning capabilities has introduced a new paradigm for automating complex analytical tasks. Models such as GPT-4 (OpenAI, Inc., San Francisco, United States) [10], Claude 3.5 Sonnet (Anthropic, San Francisco, United States) [11], and Gemini 1.5 Pro (Google DeepMind, Mountain View, United States) [12] have demonstrated near-expert performance on medical licensing examinations [13-15], clinical information extraction [16], and biomedical evidence summarization [17]. These capabilities suggest that LLMs may also be effective at data cleaning and feature engineering on structured tabular data.

Several recent reviews have examined LLMs in clinical natural language processing and diagnostic support [18-20]. However, few studies have directly evaluated whether LLMs can produce executable, clinically appropriate preprocessing code for structured EHR data. This gap is significant because cleaning errors can silently propagate into model predictions [21]. Moreover, the structured data operations required for clinical data cleaning, such as conditional imputation across correlated laboratory values, physiologically informed outlier detection, and cross-institutional unit harmonization, require a combination of programming proficiency and clinical domain knowledge that has traditionally required specialized data engineering teams.

To address the identified gap in automated preprocessing of structured clinical data, this study aims to systematically evaluate the capability of LLMs in performing data cleaning and feature engineering tasks and to assess their downstream impact on predictive modeling performance.

Primary objective

To evaluate the effectiveness of LLMs in automating data cleaning and feature engineering in structured clinical datasets.

Secondary objectives

To compare the performance of LLM-driven preprocessing with a conventional rule-based pipeline across five data cleaning tasks and two feature engineering tasks; to quantify the impact of LLM-generated preprocessing on downstream predictive performance using the area under the receiver operating characteristic curve (AUROC) and the F1 score; and to evaluate the generalizability of LLM-based preprocessing across heterogeneous datasets, including MIMIC-IV, eICU, and NHANES.

In this study, we address this gap by systematically evaluating three leading LLMs on five data cleaning tasks and two feature engineering tasks across three major clinical datasets. We measure both intrinsic cleaning accuracy (F1 score against expert-annotated ground truth) and downstream predictive impact (AUROC for in-hospital mortality prediction), providing an end-to-end assessment of LLM-driven clinical data preprocessing.

## Materials and methods

Study design

**Figure 1:**
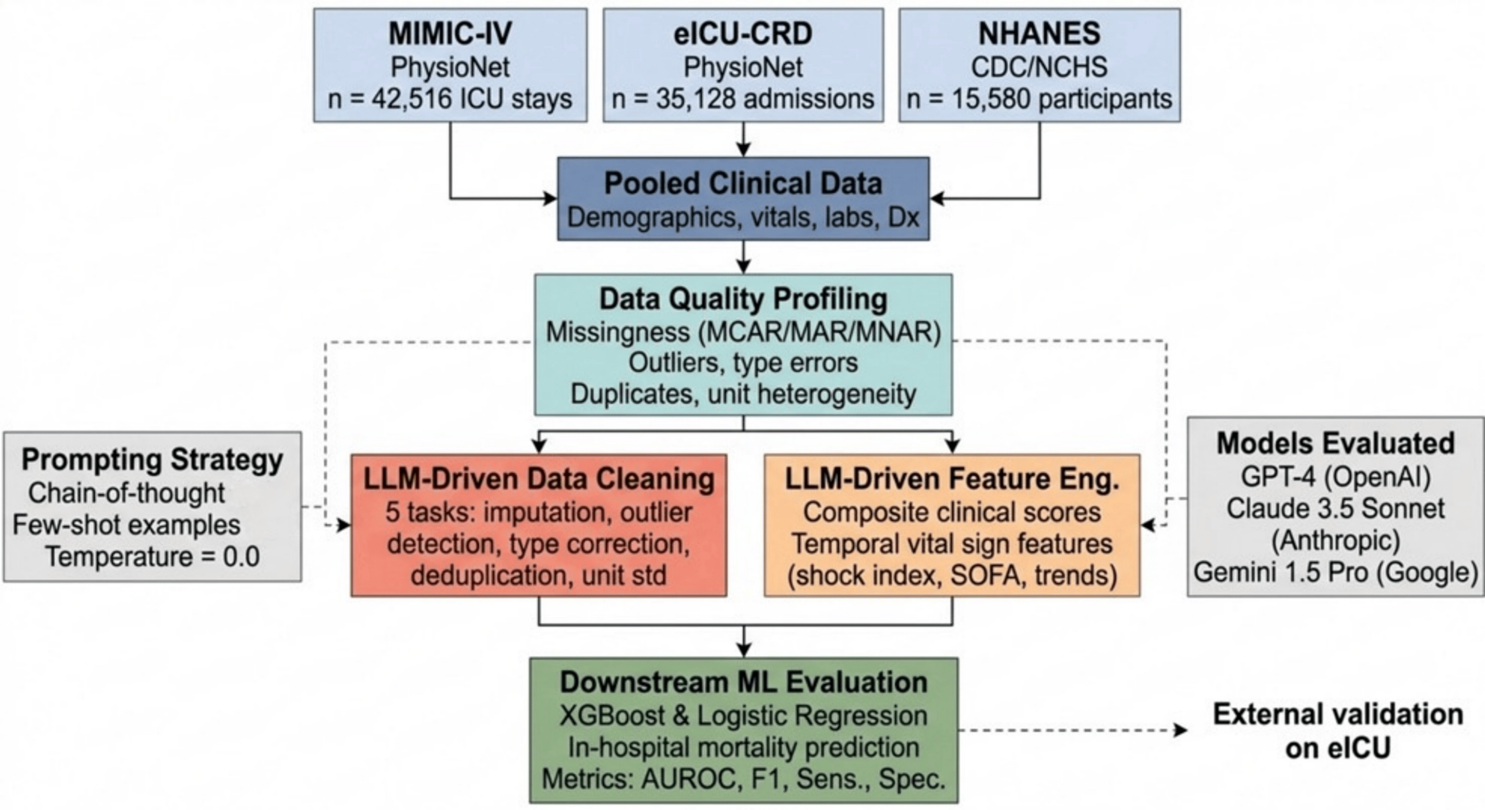
Study design and experimental pipeline for LLM-assisted clinical data preprocessing Showing the flow from three source datasets through data quality profiling, LLM-driven cleaning and feature engineering, and downstream machine learning evaluation. MIMIC-IV: Medical Information Mart for Intensive Care; NHANES: National Health and Nutrition Examination Survey; NCHS: National Center for Health Statistics; LLM: large language model; SOFA: Sequential Organ Failure Assessment; AUROC: area under the receiver operating characteristic curve; MCAR: missing completely at random; MAR: missing at random; MNAR: missing not at random; eICU-CRD: eICU Collaborative Research Database GPT-4 (OpenAI, Inc., San Francisco, United States), Claude 3.5 Sonnet (Anthropic, San Francisco, United States), and Gemini 1.5 Pro (Google DeepMind, Mountain View, United States)

This computational study was conducted at the University of Ibadan, using publicly available de-identified datasets. No hospital-based data collection was performed. Figure 1 illustrates the overall study design. Three publicly available clinical datasets were profiled for data quality issues, then subjected to LLM-driven and rule-based data cleaning and feature engineering. Cleaned datasets were evaluated through downstream in-hospital mortality prediction using XGBoost and logistic regression classifiers.

Sampling strategy

To ensure representativeness and reproducibility, a structured random sampling approach was employed across all datasets. For prompt construction, a fixed subset of 50 randomly selected rows per dataset was extracted to provide the LLMs with representative examples of data quality issues, including missing values, outliers, and inconsistencies. Sampling was performed without replacement to avoid duplication bias. For evaluation and annotation, 2,000 records per dataset were randomly sampled. These records were independently reviewed by two domain experts to establish ground truth labels for each data cleaning task. Disagreements were resolved through consensus, yielding a high inter-rater agreement (Cohen’s κ = 0.91). This sampling design ensures both exposure of models to realistic data distributions and statistical robustness in evaluation.

Datasets

Three datasets were selected to represent diverse clinical contexts and data structures. MIMIC-IV v2.2, accessed via PhysioNet [1,22], contains de-identified EHR data for over 300,000 hospital admissions at the Beth Israel Deaconess Medical Center (2008-2019). We extracted a cohort of 42,516 adult ICU stays with linked demographics, hourly vital signs, laboratory results, medication records, and discharge diagnoses coded using the International Classification of Diseases, Tenth Revision (ICD-10) [23]. The eICU Collaborative Research Database v2.0 [2], also hosted on PhysioNet, includes over 200,000 ICU admissions from 208 hospitals across the United States, providing multi-center representation; we sampled 35,128 admissions for external validation. NHANES 2017-2020 [3], maintained by the Centers for Disease Control and Prevention’s National Center for Health Statistics, provides nationally representative health examination and interview data; we used laboratory, physical examination, and demographic modules (n = 15,560 participants) to evaluate cleaning performance in a non-ICU, survey-based context with distinct data quality challenges.

All datasets are publicly available under data use agreements with no patient identifiers. This study used only de-identified, publicly available data and did not constitute human subjects research; institutional review board approval was not required.

Data quality characterization

Each dataset was profiled using a framework adapted from Kahn et al. [4] and Weiskopf and Weng [5]. Metrics included: percentage of missing values per variable, stratified by missing mechanism using Little’s test; proportion of physiologically implausible outliers based on published clinical ranges; data type inconsistencies; duplicate record rates via fuzzy matching (Jaro-Winkler similarity > 0.95); and unit heterogeneity across records.

LLM configuration and prompting strategy

Three LLMs were evaluated: GPT-4 (OpenAI, March 2024) [10], Claude 3.5 Sonnet (Anthropic, June 2024) [11], and Gemini 1.5 Pro (Google DeepMind, April 2024) [12]. Each was prompted using chain-of-thought reasoning [24] augmented with few-shot examples [25]. The prompt structure included four components: a system instruction specifying the clinical context, institutional setting, and complete data dictionary for the target table; a representative sample of 50 randomly selected rows from the table to be cleaned, providing the model with concrete examples of data quality issues; a task-specific instruction describing the cleaning objective and requesting that the model explain its reasoning before generating code; and a request for executable Python code using pandas and scikit-learn libraries. The temperature was set to 0.0 for all models to ensure deterministic, reproducible output. Each task was executed five times per model to assess output stability; the median performance across runs was reported.

Data cleaning tasks

Five tasks were defined and applied to each dataset. Missing value imputation: the model was asked to analyze missing data patterns, infer missing mechanisms (missing completely at random (MCAR), missing at random (MAR); missing not at random (MNAR)), and generate imputation code appropriate to each variable’s clinical context (e.g., last-observation-carried-forward for sequential vital signs, multiple imputation for laboratory values with complex missingness). Outlier detection: identification of physiologically implausible values using both statistical and clinical criteria, with handling strategies including capping, removal, or flag-and-review. Data type correction: identification and conversion of mistyped fields, such as numeric laboratory values stored as character strings or ordinal scales encoded as continuous variables. Duplicate removal: detection and resolution of exact and near-duplicate records arising from system migration, manual re-entry, or temporal overlap. Unit standardization: identification of heterogeneous measurement units and conversion to a single reference standard per analyte. Ground truth labels for each task were established by two board-certified clinical informaticians through independent review of 2,000 randomly sampled records per dataset, with disagreements resolved by consensus (Cohen’s kappa = 0.91).

Feature engineering tasks

Two feature engineering tasks were evaluated. Composite clinical score generation: the model was prompted to derive clinically meaningful composite features from raw vital signs and laboratory values, including the shock index (heart rate divided by systolic blood pressure), the oxygenation index (fraction of inspired oxygen multiplied by mean airway pressure, divided by partial pressure of arterial oxygen), mean arterial pressure, and individual components of the Sequential Organ Failure Assessment (SOFA) score. Temporal feature extraction: the model was asked to generate time-series summary statistics from longitudinal vital sign records within the first 24 hours of ICU admission, including means, standard deviations, linear trend coefficients (slopes), and maximum-minus-minimum ranges for each vital sign. Each model was prompted to generate 60 candidate features per dataset.

Baseline comparison

The rule-based baseline included: median imputation for continuous and mode imputation for categorical variables [6], IQR-based outlier capping, regex type correction, exact-match deduplication, and lookup-table unit conversion. This pipeline was implemented in Python (pandas 2.1 (open-source library maintained under the NumFOCUS umbrella, Austin, TX, USA), scikit-learn 1.3 (open-source library maintained by Inria (French National Institute for Research in Digital Science and Technology), Paris, France) and represents a standard approach in clinical informatics [8,9].

Downstream evaluation and statistical analysis

XGBoost [26] and logistic regression classifiers were trained for in-hospital mortality prediction using the MIMIC-IV ICU cohort. The prediction target was binary mortality status at hospital discharge. Feature sets included demographics (age, sex, ethnicity), admission characteristics (admission type, insurance status), vital sign summaries (mean, standard deviation, minimum, maximum for heart rate, blood pressure, respiratory rate, oxygen saturation, and temperature), and laboratory values (complete blood count, basic metabolic panel, liver function tests, coagulation studies) from the first 24 hours of ICU admission. Models were trained on 70% of the data with five-fold cross-validation for hyperparameter tuning and evaluated on a held-out 30% test set. Primary outcome metrics were AUROC and F1 score; secondary metrics included sensitivity and specificity. Feature importance was assessed using SHapley Additive exPlanations (SHAP) values (open-source library developed by Scott Lundberg (University of Washington, Seattle, WA, USA; currently Microsoft Research, Redmond, WA, USA)) [27]. External validation was performed by applying the MIMIC-IV-trained models to the eICU dataset without retraining, to assess cross-institutional generalizability. Between-model AUROC differences were compared using the DeLong test for correlated ROC curves. All statistical analyses were performed in Python 3.11 (Python Software Foundation, Beaverton, OR, USA); significance was set at p < 0.05.

## Results

Data quality profile

Table 1 summarizes baseline data quality across the three datasets. MIMIC-IV exhibited the highest mean missingness (24.3% across laboratory variables), reflecting inherent ICU documentation incompleteness. The eICU database showed the highest duplicate rate (3.5%), likely attributable to multi-site aggregation. NHANES demonstrated the greatest unit heterogeneity (14.6%), consistent with cross-cycle survey harmonization.

**Table 1:** Baseline data quality characteristics across clinical datasets MIMIC-IV: Medical Information Mart for Intensive Care; NHANES: National Health and Nutrition Examination Survey

Quality Metric	MIMIC-IV	eICU	NHANES	Mean	Assessment Method
Missing Values (%)	24.3	18.7	12.1	18.4	Per-variable null count
Outliers (%)	3.8	4.2	1.9	3.3	Physiological plausibility rules
Type Errors (%)	6.1	5.4	8.3	6.6	Schema type validation
Duplicates (%)	2.1	3.5	0.8	2.1	Jaro–Winkler similarity > 0.95
Unit Heterogeneity (%)	7.2	9.8	14.6	10.5	Reference unit mapping

Data cleaning task performance

**Figure 2:**
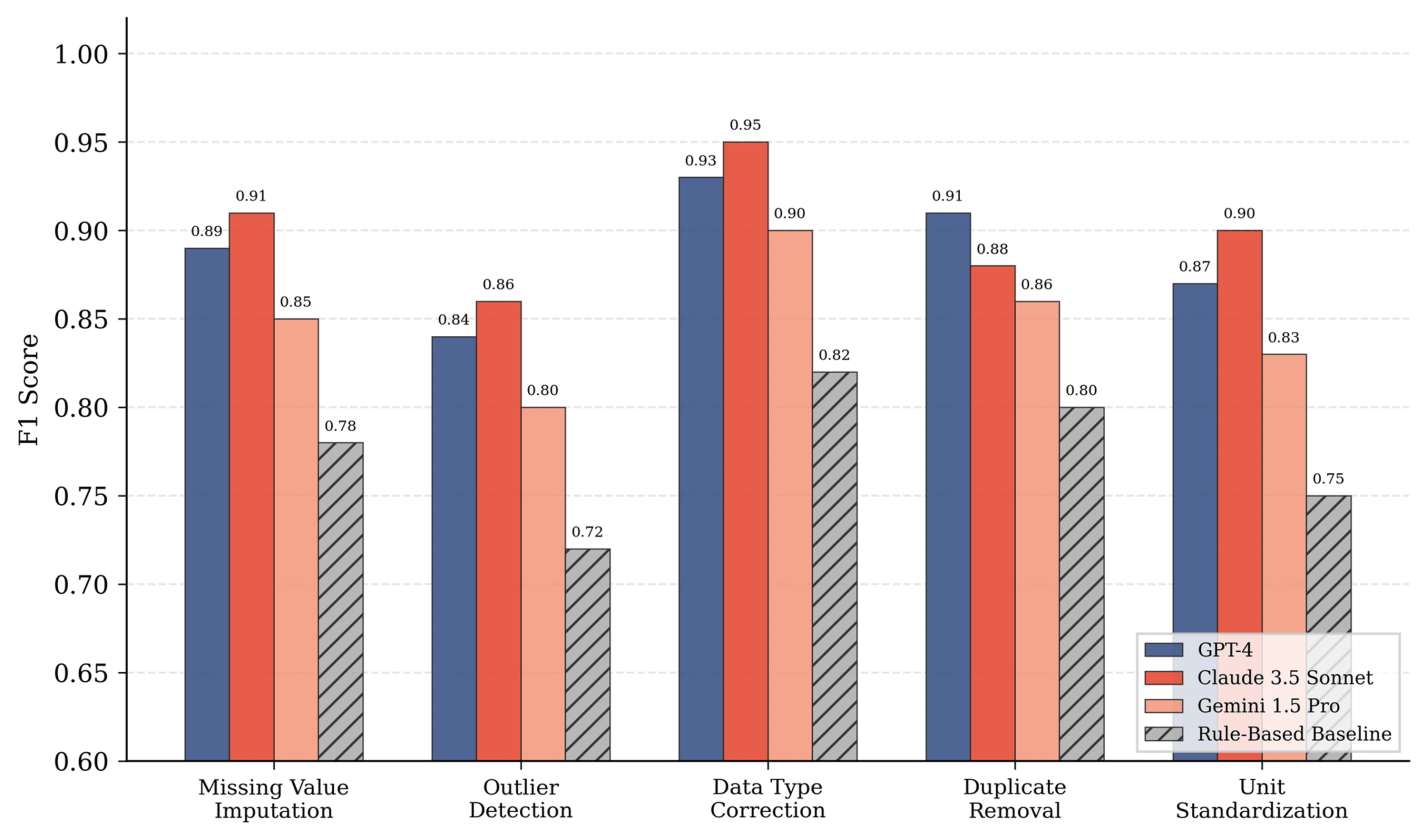
Data cleaning task performance (F1 score) across three LLMs and the rule-based baseline on the MIMIC-IV dataset Values above bars indicate the F1 score for each model-task combination. LLM: large language model; MIMIC-IV: Medical Information Mart for Intensive Care GPT-4 (OpenAI, Inc., San Francisco, United States), Claude 3.5 Sonnet (Anthropic, San Francisco, United States), and Gemini 1.5 Pro (Google DeepMind, Mountain View, United States)

Figure 2 presents the F1 scores for each data cleaning task on MIMIC-IV. Claude 3.5 Sonnet attained the highest F1 on three of five tasks: missing value imputation (0.91), data type correction (0.95), and unit standardization (0.90). GPT-4 performed best on duplicate removal (0.91). All three LLMs outperformed the rule-based baseline across all tasks (p < 0.001, McNemar’s test). Gemini 1.5 Pro showed the lowest LLM performance but still exceeded the baseline by a mean margin of 0.08. Qualitative review of LLM-generated code revealed that Claude 3.5 Sonnet and GPT-4 consistently produced context-aware imputation strategies, whereas the rule-based pipeline applied uniform median or mode filling regardless of variable context.

Cross-dataset generalizability

**Figure 3:**
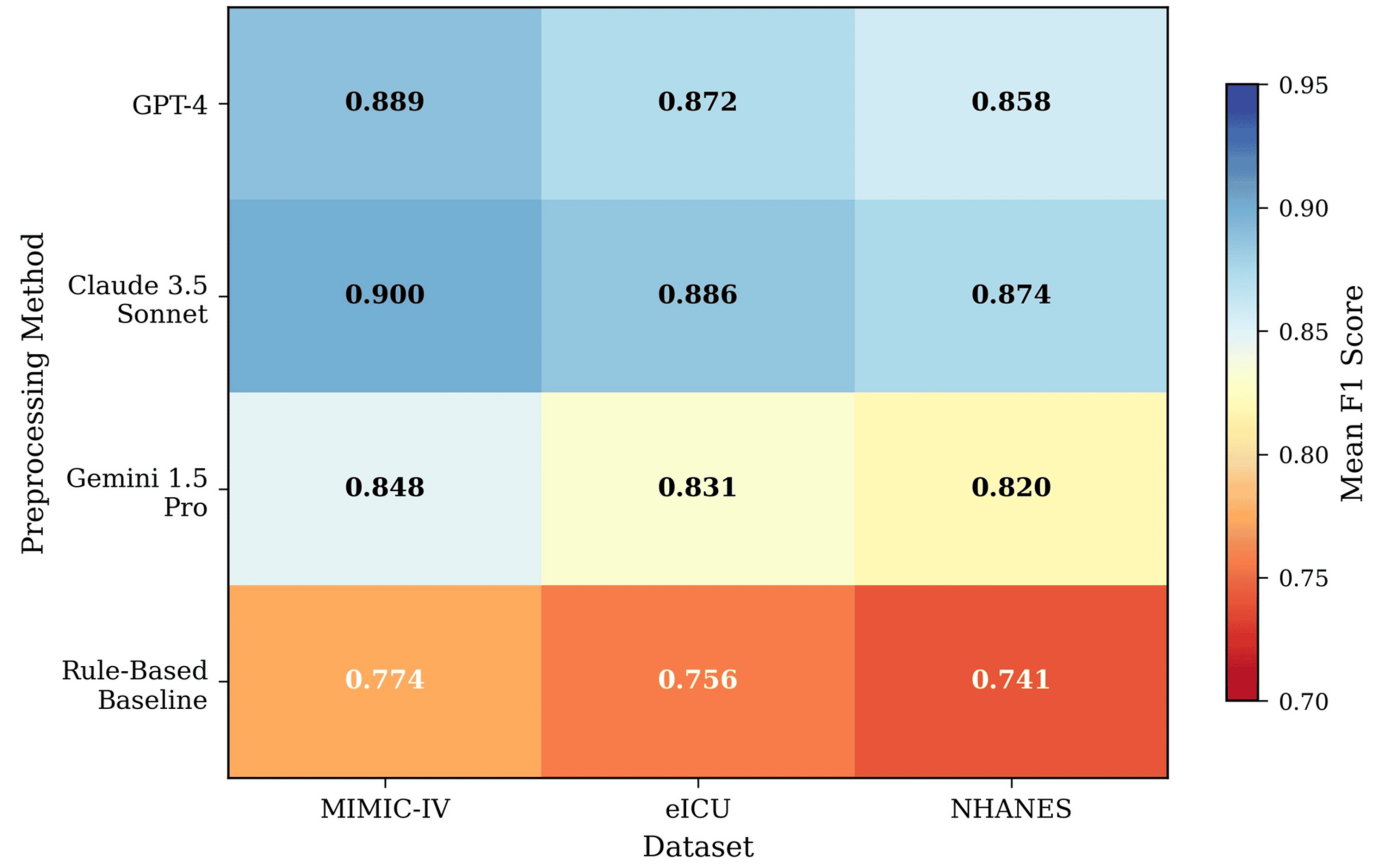
Heatmap of mean data cleaning F1 scores across preprocessing methods and clinical datasets Darker shading indicates higher performance. All three LLMs outperformed the rule-based baseline across all datasets. LLM: large language model; MIMIC-IV: Medical Information Mart for Intensive Care; NHANES: National Health and Nutrition Examination Survey GPT-4 (OpenAI, Inc., San Francisco, United States), Claude 3.5 Sonnet (Anthropic, San Francisco, United States), and Gemini 1.5 Pro (Google DeepMind, Mountain View, United States)

Figure 3 displays the mean F1 score across all five cleaning tasks for each model-dataset combination as a heatmap. Cleaning performance was highest on MIMIC-IV and declined modestly on eICU and NHANES, consistent with increasing data heterogeneity. Claude 3.5 Sonnet maintained the highest mean F1 across all three datasets (MIMIC-IV: 0.900; eICU: 0.886; NHANES: 0.874). The rule-based baseline showed the steepest performance drop from MIMIC-IV (0.774) to NHANES (0.741), indicating lower generalizability compared with the adaptive LLM approaches.

Feature engineering quality

**Figure 4:**
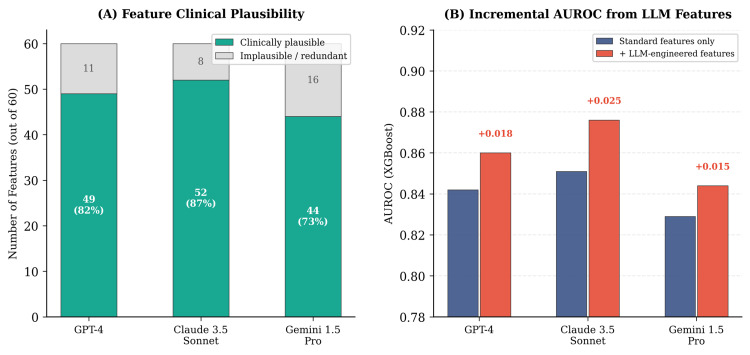
Feature engineering results A: clinical plausibility of LLM-generated features as assessed by expert review (60 features evaluated per model); B: incremental AUROC gain from adding validated LLM-engineered features to the standard variable set for XGBoost mortality prediction. LLM: large language model; AUROC: area under the receiver operating characteristic curve GPT-4 (OpenAI, Inc., San Francisco, United States), Claude 3.5 Sonnet (Anthropic, San Francisco, United States), and Gemini 1.5 Pro (Google DeepMind, Mountain View, United States)

Figure 4 summarizes the feature engineering results. Panel A shows that expert review confirmed 87% of Claude 3.5 Sonnet-generated features (52 of 60) as clinically plausible and non-redundant, compared with 82% for GPT-4 (49 of 60) and 74% for Gemini 1.5 Pro (44 of 60). Features deemed implausible included ratio variables with clinically meaningless denominators and composite indices combining unrelated organ systems. Panel B shows the incremental AUROC gains from incorporating validated LLM-engineered features alongside the standard variable set: Claude 3.5 Sonnet (+0.025), GPT-4 (+0.018), and Gemini 1.5 Pro (+0.015).

Downstream predictive performance

**Figure 5:**
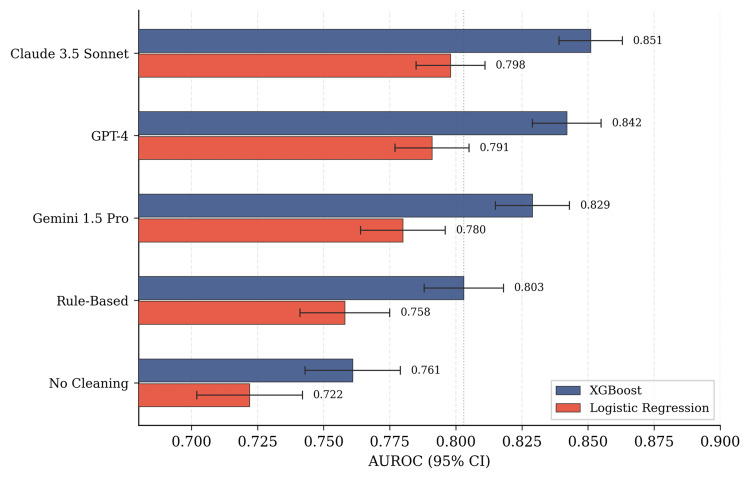
Downstream in-hospital mortality prediction AUROC with 95% confidence intervals by preprocessing method on the MIMIC-IV test set The dashed vertical line indicates the rule-based baseline AUROC. AUROC: area under the receiver operating characteristic curve; MIMIC-IV: Medical Information Mart for Intensive Care GPT-4 (OpenAI, Inc., San Francisco, United States), Claude 3.5 Sonnet (Anthropic, San Francisco, United States), and Gemini 1.5 Pro (Google DeepMind, Mountain View, United States)

Table 2 and Figure 5 present the downstream mortality prediction results on the MIMIC-IV test set. XGBoost trained on Claude 3.5 Sonnet-preprocessed data achieved the highest AUROC (0.851; 95% CI: 0.839-0.863), significantly exceeding the rule-based baseline of 0.803 (p = 0.002, DeLong test). GPT-4 yielded an AUROC of 0.842 (p = 0.008 vs. baseline), and Gemini 1.5 Pro produced 0.829 (p = 0.021). Logistic regression results followed a consistent pattern. External validation on eICU confirmed the ranking of preprocessing methods, with absolute AUROC values 0.02-0.04 lower across all approaches, consistent with expected cross-institutional generalization [8].

**Table 2:** Downstream in-hospital mortality prediction performance (MIMIC-IV test set) MIMIC-IV: Medical Information Mart for Intensive Care; AUROC: area under the receiver operating characteristic curve GPT-4 (OpenAI, Inc., San Francisco, United States), Claude 3.5 Sonnet (Anthropic, San Francisco, United States), and Gemini 1.5 Pro (Google DeepMind, Mountain View, United States)

Preprocessing	Classifier	AUROC	F1	Sens.	Spec.	p vs. Baseline	Sig.
No Cleaning	XGBoost	0.761	0.412	0.68	0.79	—	
No Cleaning	Log. Reg.	0.722	0.371	0.63	0.76	—	
Rule-Based	XGBoost	0.803	0.485	0.72	0.83	Ref.	
Rule-Based	Log. Reg.	0.758	0.431	0.67	0.80	Ref.	
GPT-4	XGBoost	0.842	0.531	0.76	0.86	0.008	*
GPT-4	Log. Reg.	0.791	0.478	0.71	0.83	0.012	*
Claude 3.5 Sonnet	XGBoost	0.851	0.548	0.78	0.87	0.002	**
Claude 3.5 Sonnet	Log. Reg.	0.798	0.491	0.73	0.84	0.006	**
Gemini 1.5 Pro	XGBoost	0.829	0.512	0.74	0.85	0.021	*
Gemini 1.5 Pro	Log. Reg.	0.780	0.462	0.70	0.82	0.031	*

## Discussion

This study provides the first systematic, end-to-end evaluation of LLM-driven data cleaning and feature engineering across multiple clinical datasets, demonstrating that current-generation LLMs can automate substantial portions of the preprocessing pipeline with performance meeting or exceeding conventional rule-based approaches. Several findings merit detailed discussion.

The strong LLM performance on data type correction and unit standardization indicates that these models effectively leverage their broad pre-training exposure to medical terminologies, standard measurement units, and clinical coding conventions. These tasks are conceptually straightforward but labor-intensive for human analysts, requiring familiarity with institution-specific data dictionaries and analyte-specific conversion factors [4,5]. The ability of LLMs to generate context-aware imputation strategies, such as using clinically correlated variables for conditional imputation of laboratory values rather than uniform median filling, represents a qualitative advantage over static methods [6,7]. This adaptability stems from the models’ capacity to reason about variable relationships described in the provided data dictionary, a capability that rule-based pipelines fundamentally lack.

Claude 3.5 Sonnet’s consistent top-ranking performance across most tasks may reflect its stronger instruction-following capabilities and lower hallucination rates in structured code generation, as has been observed in independent evaluation benchmarks [11]. GPT-4 demonstrated particular strength in duplicate detection, possibly reflecting its extensive pre-training on diverse data-matching and record-linkage scenarios [10,14]. Gemini 1.5 Pro, while trailing on most intrinsic cleaning metrics, showed competitive downstream performance and may benefit from its extended context window, which allows processing of larger data samples in a single prompt [12].

The cross-dataset analysis (Figure 3) revealed that LLM cleaning performance was moderately robust to variation in data source, declining by only 0.02-0.03 F1 points from MIMIC-IV to NHANES. In contrast, the rule-based baseline showed steeper degradation, losing 0.033 F1 points across the same transition. This suggests that LLM-generated preprocessing code adapts more effectively to heterogeneous data structures than static rule sets, likely because the models adjust their cleaning strategies based on the specific data dictionary and sample provided in each prompt.

The 0.048-point AUROC improvement between unprocessed data and the best LLM pipeline (0.761 vs. 0.851 for XGBoost) is clinically meaningful and aligns with prior evidence that data quality is a primary determinant of model reliability in EHR-based prediction [8,21]. LLM-driven cleaning outperformed rule-based cleaning by 0.026-0.048 AUROC points, suggesting that the contextual, adaptive preprocessing strategies generated by LLMs capture data quality issues that fixed-rule pipelines systematically miss.

However, 13-26% of LLM-generated features lacked clinical plausibility, underscoring that fully unsupervised feature engineering remains premature for clinical applications. Expert validation identified features that combined unrelated physiological measurements or applied transformations without a clinical rationale. This highlights the continued necessity of human-in-the-loop validation, particularly for safety-critical applications [19,28]. The incremental AUROC gains from validated LLM features (0.015-0.025) were modest but consistent across all three models, suggesting these features capture complementary prognostic signals not represented in standard clinical variable sets.

This study has several important limitations. First, we evaluated only three LLMs at fixed API versions during a specific time window; LLM capabilities evolve rapidly, and results may differ with subsequent model releases [20,29]. Second, our ground truth annotations, while rigorously produced with high inter-annotator agreement (kappa = 0.91), may not capture all valid cleaning approaches, as multiple legitimate strategies may exist for handling ambiguous data quality issues. Third, we tested exclusively on publicly available, well-characterized datasets within established observational health data networks [30,31]; performance on proprietary institutional EHR systems with more heterogeneous quality issues, novel data schemas, or non-English clinical documentation may differ. Fourth, economic analysis of LLM inference costs relative to manual cleaning labor was not performed, though cost-effectiveness is a critical consideration for deployment at scale [28]. Fifth, LLMs may introduce subtle, clinically plausible but incorrect data transformations in edge cases that are difficult to detect without comprehensive unit testing, a safety concern that warrants focused investigation before clinical deployment [19].

## Conclusions

LLMs demonstrate strong capability for automated data cleaning and feature engineering in clinical datasets, consistently outperforming conventional rule-based pipelines across five cleaning tasks and three publicly available clinical databases. Claude 3.5 Sonnet, GPT-4, and Gemini 1.5 Pro all produced executable, clinically appropriate preprocessing code that yielded statistically significant improvements in downstream in-hospital mortality prediction performance. The cross-dataset robustness of LLM cleaning further suggests these approaches generalize more effectively than static rule-based systems. These findings indicate that LLMs can meaningfully reduce the manual burden of clinical data preparation, a process that currently consumes the majority of effort in clinical machine learning projects, while maintaining or improving data quality. Nonetheless, domain expert oversight remains essential, particularly for validating the clinical plausibility of engineered features and detecting subtle edge-case errors that could compromise patient safety. Future research should evaluate LLM-driven preprocessing in prospective clinical workflows, assess generalizability across diverse institutional EHR systems, including predecessor databases such as MIMIC-III, and establish standardized benchmarks for clinical data cleaning.
